# AlignMarkers: an original pipeline for placing molecular markers on physical sequences

**DOI:** 10.1186/s12859-026-06582-1

**Published:** 2026-08-06

**Authors:** Camille Auneau, Baptiste Imbert, Mathieu Zemihi, Grégoire Aubert, Nadim Tayeh, Jonathan Kreplak

**Affiliations:** Université Bourgogne Europe, Institut Agro Dijon, INRAE, Agroécologie, Dijon, France

**Keywords:** Single nucleotide polymorphism (SNP), Insertion-deletion (indel), Genomic context sequence, Nucleotide alignment, Assembly-to-assembly marker position transfer, Nextflow

## Abstract

**Background:**

Continuous progress in genome sequencing and assembly, coupled with the growing availability of massive resources of long-read genomic sequences and high-quality molecular markers, based on single nucleotide polymorphisms (SNPs) and insertions-deletions (indels), demands accurate methods to easily, accurately and readily determine marker positions across genome versions. This is particularly important for applications such as development and updating of genotyping array. However, existing tools often require an associated reference genome for the molecular markers and additional adaptations are needed to map the short context sequences of these markers when no such reference genome is available. To overcome these limitations, our aim was to develop an original pipeline.

**Results:**

AlignMarkers is a robust bioinformatics pipeline designed to accurately place molecular markers on genome assemblies without requiring information on initial positions on a reference genome, using sequence alignment. It can also operate on coordinate-based files to perform liftover-like analyses, providing an alternative when genome-to-genome alignments could not be generated. It accepts multiple input file formats (VCF, BED, FASTA, CSV) and is optimized for sequences of  ≥ 100 base pairs. Context sequences are retrieved, when only coordinates are provided, and then aligned to target genomes using Minimap2, followed by a stringent filtering process ensuring alignment uniqueness, high sequence identity, and verification of the expected nucleotide. AlignMarkers is built with Nextflow and Python and integrates established tools such as Minimap2, Samtools, and Bedtools. It generates comprehensive reports and visualizations to facilitate result interpretation. We evaluated AlignMarkers using 80,000 randomly selected positions across the version 2 *Pisum sativum* genome assembly of cultivar Cameor, showing that increasing flanking sequence length improves placement accuracy and reduces multimapping, especially in repeat-rich regions. We further benchmarked AlignMarkers on 100,000 *Solanum lycopersicum* SNPs and indels against CrossMap and bcftools/liftover, showing high concordance in the outputs of all tools. These results demonstrate the robustness and reliability of the pipeline when used with different marker resources.

**Conclusion:**

AlignMarkers is a reliable and user-friendly solution for transferring marker positions. It supports multiple applications and facilitates the management of large sets of molecular markers for various purposes, including the construction of genotyping platforms.

**Supplementary Information:**

The online version contains supplementary material available at https://doi.org/10.1186/s12859-026-06582-1.

## Background

The quality and quantity of genome assemblies have improved rapidly in recent years [1] due to continual advances in sequencing technologies, coupled with a progressive and significant drop in sequencing costs [2, 3]. Consequently, there is a growing need for reliable and robust tools to transfer information from initial genome assemblies to more recent releases. This is especially true for molecular markers. Based on single nucleotide polymorphism (SNP) or insertion-deletion (indel) events, molecular markers are instrumental in understanding the genetic architecture of key traits and represent great tools for population genetic studies, marker-assisted breeding and genomic selection programs [4].

CrossMap [5], UCSC LiftOver [6, 7] and Bcftools/Liftover [8] are three widely used tools for converting genomic coordinates and annotation files between different assemblies. They rely on genome-to-genome alignment files known as chain files, which encode the correspondence between regions of different genome assemblies [9]. CrossMap supports multiple file formats, including BAM [10], CRAM [11], SAM [10], BED [12], GFF [13], GTF [14] and VCF [15], whereas UCSC liftOver supports BED, GFF and GTF files, and BCFtools/liftover is restricted to VCF/BCF files.

However, their performance depends on the quality and availability of chain files, which only capture conserved blocks and therefore require a sufficient degree of sequence similarity between assemblies to ensure reliable coordinate conversion.

Furthermore, although precomputed chain files are available on the UCSC website [7], these are primarily provided for model organisms (e.g. human, mouse and zebrafish) and reference genomes. For other species, users must generate their own chain files. Although several pipelines [16] and tools [17–19] exist to automate the production of chain files, large and repeat-rich genomes could still present a challenge [14] in terms of computing resources for alignments.

In addition, these tools show reduced accuracy in complex regions, such as those rich in repetitive elements or carrying large indels [20–23]. According to the UCSC FAQ [24], LiftOver is not recommended for converting SNPs between assemblies because very short context sequences as for SNPs may not achieve a sufficiently high alignment score to be considered successfully converted. They advise using SNP tracks or rsIDs to map variants to the target assembly, possibly combining this approach with LiftOver for variants that cannot be mapped directly. They are also unsuitable for markers that are only known by their sequences, for which there are no source coordinates with which to perform the conversion.

To overcome the above-mentioned limitations, we developed AlignMarkers, a pipeline specifically designed for the accurate remapping of molecular markers (SNPs and indels) based on their context sequences across genome assemblies of the same cultivar or species or beyond. The pipeline relies on genomic alignments performed with Minimap2 to map SNPs and indels onto a target genome. A careful verification step ensures the reliability of each alignment by evaluating the exact positioning of variants’ nucleotides, checking that variants match the expected nucleotides, detecting multimapping, and providing a percentage identity.

The pipeline offers high flexibility in terms of input formats including VCF, BED, CSV and FASTA [25], and can process multiple files simultaneously, which facilitates handling markers from heterogeneous sources, including those lacking initial genome coordinates or originating from different genome assemblies. It is particularly useful for integrative approaches that aim to bring together all available knowledge within a single framework, for example when designing genotyping arrays or enabling the transfer of genetic information across species.

Furthermore, AlignMarkers offers a range of features designed to streamline user tasks. For example, it can extract context sequences for target markers even when only variant positions are provided. It also allows cross-species markers to be highlighted by aligning their context sequences simultaneously across different species’ genomes. In such cases, each genome is first processed independently, with all chromosome identifiers being uniformly renamed with a species or genome suffix (e.g. chr1_Species1, chr2_Species1, chr1_Species2). This prevents naming conflicts between equivalent chromosomes from different species, allowing them to be identified unambiguously once the genomes are concatenated into a single reference.

In summary, AlignMarkers’ philosophy is to allow markers from various sources and genome assemblies, including large and complex genomes, to be mapped accurately onto a target reference. This is a useful, practical and reliable addition to the bioinformatics toolbox for researchers.

## Implementation

AlignMarkers is a bioinformatics pipeline designed to accurately position molecular markers (SNPs and indels) on different versions of genome assemblies. It can accept one or multiple input files containing information about the markers of interest. These files can be in various formats, including VCF, BED, FASTA and CSV.

In accordance with Minimap2’s recommendations, the pipeline has been optimised for sequences of a total length of at least 100 bp, which ensures reliable alignment from this minimum length [26]. Below this threshold, Minimap2 may fail to align sequences accurately.

To ensure robust results, a filtering step removes low-quality alignments based on specific criteria. Accompanying visualisations and detailed reports provide clear insights to facilitate thorough analysis.

### Data input

AlignMarkers accepts two types of marker input files. The first type, ‘Sequence input’, contains two elements: the marker identifier and the corr esponding context sequence. For each marker, the polymorphism (SNP or indel) is indicated between square brackets. This information can be contained in a FASTA file as shown below:

> INDEL1

ATTCGA[T/ATTTT]GCAC

Or in a CSV file as following:

INDEL1,ATTCGA[T/ATTTT]GCAC

The second type of marker file, ‘Positional input’, includes the following mandatory fields: the marker identifier, the respective chromosome identifier, the position of the marker in base pair on the chromosome and the reference and alternative alleles, as exemplified below in CSV format:

SNP1,Chr5,548,312,A,T

In the latter case, the FASTA file of the original reference genome (i.e. the genome in which the coordinates were defined) must be provided in order to retrieve the context sequences relative to the markers and align them on the new, target genome.

For both types of marker files, it is required to provide the target genome in FASTA format whether gzipped or not.

### Sequence preparation

**Fig. 1:**
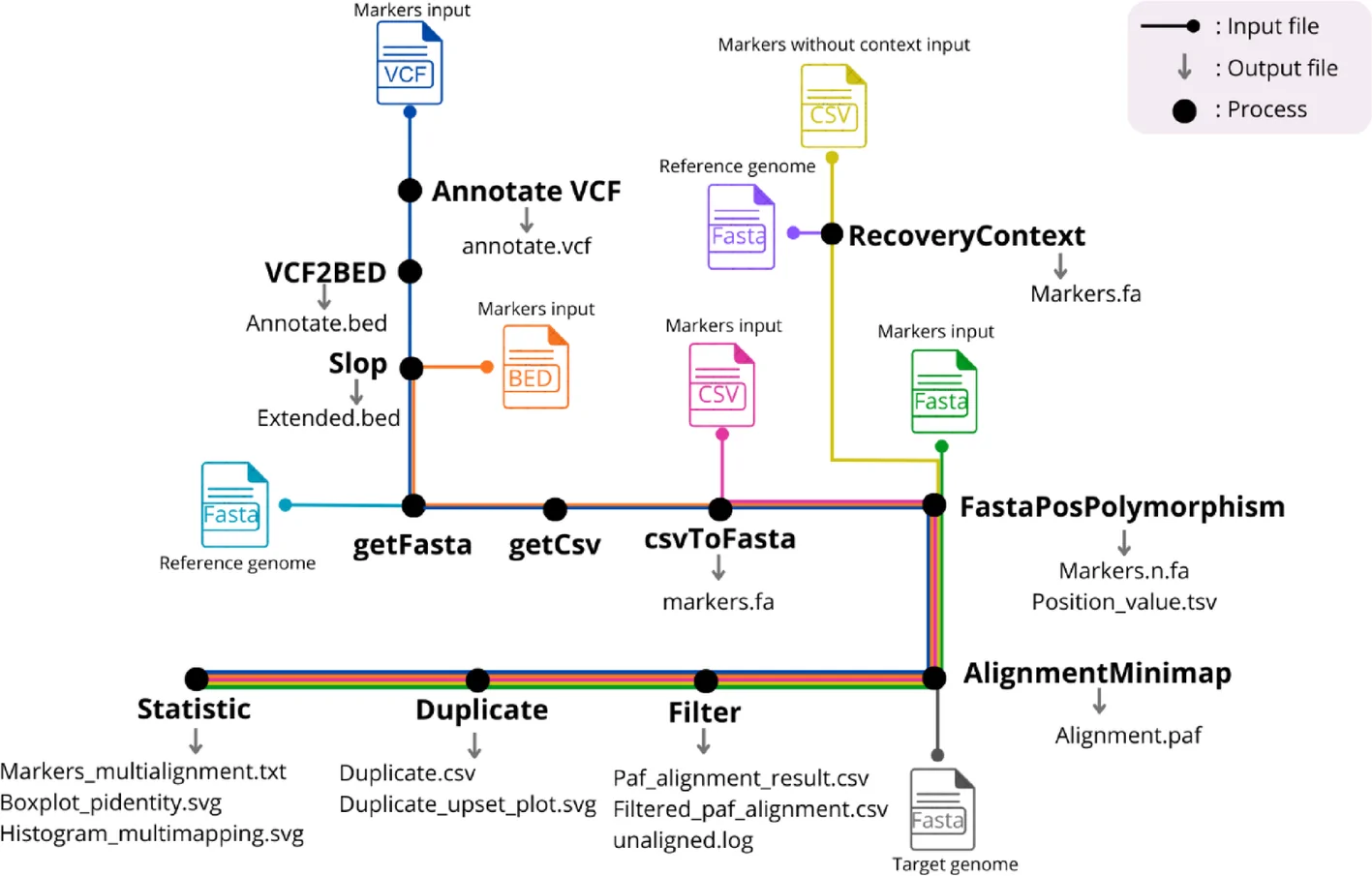
Detailed workflow of the AlignMarkers pipeline

The input data undergoes a series of pre-processing steps to ensure consistent formatting, representation of variants and accurate localization of polymorphism positions relative to the reference genome prior to alignment. For example, information from BED and VCF files is used to retrieve and store the context sequence in a CSV file. This pre-processing corresponds to the steps from ‘Annotate_VCF’ to ‘csvToFasta’ in Fig. 1.

The same applies to Positional input CSV files, for which an additional step is required. This step corresponds to the ‘RecoverContext’ step (Fig. 1).

Once the context sequences have been extracted and stored in CSV files, they are converted to the FASTA format to ensure uniform processing. The FASTA sequence format is designed to include variants by inserting them directly into the context sequence between square brackets (‘[…]’). This step corresponds to the ‘csvToFasta’ step in Fig. 1. Depending on the nature of the variation (SNP or Indel), the pipeline prepares the sequences for alignment in the “FastaPosPolymorphism” step.

For SNPs, the polymorphic bases are replaced by ‘N’. This substitution enables Minimap2 to align the sequences, despite the fact that it does not explicitly handle polymorphisms encoded between square brackets.

For Indels, two sequences are generated for each marker to explicitly represent both allelic states. One sequence, labelled with the ‘_before’ suffix, corresponding to the first state of the indel representation within the bracket notation (e.g. REF in a [REF/ALT] configuration such as [A/ATT]), while the second sequence, labelled ‘_after’ corresponds to the second state (ALT in the same example, i.e. ATT). Importantly, the order of the two allelic variants within the bracket notation is interchangeable, as it does not affect the alignment outcome or the mapping results. This process enables the pipeline to align both forms and verify the presence of either allele in the target genome. The additional suffix also prevents the sequences from being incorrectly scored as multimapped.

### Sequence alignment

The prepared sequences are aligned to the target genome using Minimap2, resulting in a Pairwise mApping Format (PAF) output file. This file contains key alignment details, including the Compact Idiosyncratic Gapped Alignment Report (CIGAR) string, the target chromosome and strand, the alignment length, and the genomic position.

The CIGAR string encodes information about the alignment between the query sequence and the genome, including details of matches, insertions, deletions and substitutions. In Minimap2, both long and short CIGAR representations can be generated depending on the output format. In our pipeline, we use the long CIGAR representation, which requires enabling a specific output option (–cs = long). For instance, the CIGAR string = ATTG + ac = TAGG-a = GG*at = ATTGC indicates an insertion (+ ac), a deletion (− a), and a substitution (*at) within the aligned region. This notation enables users to verify that the detected polymorphism is located at the expected position and corresponds to the expected variant type.

### Data output

The pipeline produces a variety of output files that provide a comprehensive overview of the process and its results. These files should enable users to quickly evaluate the quality of the alignments across the entire dataset. FASTA-formatted sequences used for the alignments, along with a file listing all markers that successfully passed the filters are provided. A summary table in CSV format containing the following information for each input marker is also made available: identifier, initial position on the reference genome, aligned position on the target genome, target chromosome, alignment direction, identity score, and number of substitutions and indels observed within the context sequence frame. Additional details and descriptions of all CSV file columns are available in the project directory’s README file. This table provides a comprehensive summary of the transfer quality and enables users to quickly assess the robustness of the outputs.

Several log files are also created to report any problems encountered. For example, these could indicate that a marker does not align at any locus in the genome, or that the polymorphism is incorrect or absent.

Lastly, Nextflow [27] produces a full HTML report to provide an overview of the pipeline’s performance. This includes information such as execution time for each process step, the number of threads used, and CPU resource occupancy.

### Output filtering

Each sequence alignment is evaluated to ensure only high-confidence mappings are retained. Using the alignment information from the PAF file, ambiguous, multiple, or low-quality matches are filtered out.

For SNP markers, an alignment is considered acceptable when the polymorphic nucleotide is located at the expected position on the target genome and the observed base at this position matches any of the expected bases defined for the marker, regardless of whether it is designated as the reference or alternative base. The percentage of identity must also exceed a configurable threshold (default: 95%).

For Indel markers, only the percentage of identity is evaluated, as the exact position and the observed bases cannot be determined.

Once all the alignments have been evaluated, the pipeline applies a multimapping filter at the marker level. Only markers with a single alignment that passes all the criteria are retained; markers with multiple acceptable alignments are considered ambiguous, and no position information is provided for them. If the polymorphism is not in the expected position within the context sequence, a ‘bad position’ tag is generated, and if the polymorphism is not the expected one, a ‘bad variation tag’ is generated. This procedure ensures that only unambiguous, high-confidence marker mappings are outputted.

Example (SNP marker):

*FASTA file:*

*> SNP1*

*ATTGGA[A/C]TTAGGA*

*Polymorphism is expected at position 7, with either A or C as the expected base.*

*Expected alignment result:*

 = ATTGGA*an = TTAGGA.

With « = » indicating identical bases, «*» representing a substitution.

*Observed alignment result:*

SNP1: = ATTGGA*gn = TTAGGA → filtered out due to an unexpected nucleotide base (an observed G base, when an A or C base is expected).

SNP1: = A*anTGGATTAGGA → filtered out due to a bad position (observed polymorphism at position 2 instead of 7).

SNP1: = ATTGGA*an = TTAGGA → passes individual filters.

SNP1: = ATTGGA*cn = TTAGGA → passes individual filters.

Although each of the last two alignments passes the variation, position and identity filters individually, they are considered as multimapping. This is because the original sequence maps to two different positions on the genome. Consequently, these alignments are flagged as multimapping in the ‘*_paf_alignment_result.csv’ summary table, rather than being reported as valid hits.

Nevertheless, users can inspect the alignment file and the summary table generated by the pipeline, which contains alignment metrics such as alignment length, number of SNPs and percentage identity. They can then manually select the most relevant hit.

### Duplication identification

The pipeline incorporates a comparison step to detect redundant markers present in the input files. This involves identifying markers located in the same positions on the target genome, which indicates redundancy. This feature is particularly useful when integrating datasets from different studies, as it helps avoid duplication. The results are compiled into an output file listing all redundant markers and their associated input files, along with relevant metadata, such as position and chromosome. An UpSet plot is also generated to visualise the number of redundant markers shared between different file groups.

### Visualization

AlignMarkers produces several visualisations to aid the interpretation of the alignment results. These include the number of alignments per chromosome, the distribution of multimapped markers (for example, the number of markers mapping to two, three, or more locations) and the proportion of alignments retained or excluded by each filtering criterion. The pipeline also compares the percentage of identity values between filtered and unfiltered datasets.

### Alignment option

By default, the pipeline uses Minimap2 with the following parameters: -x sr -k15 -w10 –cs = long –secondary = yes. These settings are used to (i) ensure generation of the long CIGAR string, which is required for all downstream filtering steps, (ii) optimise alignment of relatively short context sequences (100-500 bp) for which the pipeline is designed, and (iii) retain secondary alignments, which are essential for identifying multimapped markers during repositioning analysis. In addition to these default settings, the pipeline provides optional alignment parameters to adapt the analysis to specific use cases.

A truncated alignment option allows users to define the maximum number of permitted unaligned bases at the start of the sequence. This option is evaluated on the aligned query sequence, independently of whether Minimap2 aligns the forward strand or its reverse complement and is used to correct the offset observed in some alignments. However, allowing too many unaligned bases increases the risk of multimapping and the likelihood that sequence fragments will align to several different parts of the genome.

In the case of Expressed Sequence Tags (ESTs), it is essential to adjust the Minimap2 alignment parameters especially for intron-flanking context sequence. The splice option in Minimap2 is a special mode in which long deletions will be interpreted as introns (refer to the Minimap2 manual for more information). The pipeline is adapted to integrate this option and adjust the results. Nevertheless, this option must be used sparingly. If the sequences are not ESTs, the Minimap2 setting would allow for large gaps, which may distort the alignment and the position of polymorphisms on the genome. It may also increase multimapping by allowing a greater number of potential alignments.

### Software used

AlignMarkers uses several bioinformatics tools to perform its tasks. Minimap2 [26] is employed for aligning flanking sequences on the target genome. Samtools [10] is used to index FASTA files with the *faidx* command. Bedtools [28] is used to manipulate genomic coordinates in VCF and BED files. All these tools operate within a Python environment (3.10) [29], which is distributed and executed in a Singularity-based environment [30] to ensure portability and reproducibility, that leverages multiple specialised libraries. Biopython [31] is used for sequence processing, Polars [32] for manipulating tabular data and Matplotlib [33] and R [34] with the package complexUpset [35] are used for generating visualisations. The following versions of key tools and modules are used in the current version of AlignMarkers: Minimap2 (2.26), Polars (1.30.0), Matplotlib (3.10.0), Samtools (1.17), R (4.3.3), Biopython (1.85) and Bedtools (2.31.0).

Artificial intelligence (AI) tools (ChatGPT, OpenAI) [36] were occasionally used to assist development of AlignMarkers pipeline, debugging and re-factoring some Python functions into three scripts (filter_alignment.py, paf_parsing.py, parse_table.py).

This diagram illustrates the various steps of the AlignMarkers pipeline, from preparing the input files to producing the final results. These steps include converting input formats (VCF, BED, CSV and FASTA), retrieving context sequences and aligning them with Minimap2, as well as filtering the raw alignment data. Duplicates are also identified in the input files. The output files produced at each step facilitate the analysis, quality control and visualisation of the results.

## Results

To demonstrate the effectiveness of the new pipeline and evaluate the quality of its output, several use cases were considered using datasets from various plant species. SNP and indel markers were included, as well as markers identified directly on transcribed sequences or genomic sequences. The performance of AlignMarkers was also compared to that of CrossMap and Bcftools/Liftover, two largely used tools in the research community. All analyses were run on the IFB Core computing cluster [37].

### Example application 1 based on random 80,000-marker set selected on *Pisum sativum* Cameor V2 genome assembly

A total of 80,000 nucleotide positions (Table 1), evenly distributed in untranslated regions (UTRs), repeat elements, coding sequences (CDSs) and introns, were randomly selected from the Cameor V2 *P. sativum* (pea), a large (~ 3,9 Gb) and rich in repeated sequences (~ 76%) genome [38]. These positions were distributed across all seven chromosomes, as well as unassembled scaffolds, with comparable numbers of markers observed among chromosomes for each genomic category (Supplementary Table 1). Context sequences of variable sizes were generated around these positions and AlignMarkers was used to determine the best placement of each of these positions. The approach aimed at evaluating whether specific genomic regions, such as repeat-rich regions, are associated with reduced mapping specificity or detection efficiency.

**Table 1:** Evaluation of successful placement with AlignMarkers of randomly-selected, genome-wide distributed 80,000 *Pisum sativum* markers, depending on the length of the context sequences and the nature of the host genomic regions

Flanking sequence length	Alignment category	Genomic position				TOTAL	%
		UTR	CDS	Intron	Repeat		
50	Pass, uniquely mapped	15,315	14,312	14,941	9413	53,981	67.48
	Pass, multimapped	4486	5550	4901	7351	22,284	27.86
	Fail, bad location multimapped	194	137	140	2339	2810	3.51
	Fail, bad location uniquely mapped	0	0	0	0	0	0
	Unaligned	9	1	18	897	925	1.,15
100	Pass, uniquely mapped	15,469	14,667	15,046	11,739	56,921	71.14
	Pass, multimapped	4463	5273	4898	7760	22,394	28
	Fail, bad location multimapped	67	59	51	408	585	0.73
	Fail, bad location uniquely mapped	0	0	0	0	0	0
	Pass, uniquely mapped Pass, multimapped	1	1	5	93	100	0.13
200	Pass, uniquely mapped	15,887	15,116	15,312	13,954	60,269	75.33
	Pass, multimapped	4091	4865	4672	5977	19,605	24.52
	Fail, bad location multimapped	22	18	15	64	119	0.15
	Fail, bad location uniquely mapped	0	0	0	0	0	0
	Pass, uniquely mapped Pass, multimapped	0	1	1	5	7	0.0008
500	Pass, uniquely mapped	16,605	15,881	15,993	15,327	63,806	‍79.76
	Pass, multimapped	3395	4117	4006	4659	16,177	20.22
	Fail, bad location multimapped	0	1	1	14	16	‍0.02
	Fail, bad location uniquely mapped	0	0	0	0	0	0
	Pass, uniquely mapped Pass, multimapped	0	1	0	0	1	‍0.001
1000	Pass, uniquely mapped	17,356	16,627	16,687	16,229	66,899	‍83.63
	Pass, multimapped	2644	3373	3313	3771	13,101	16.37
	Fail, bad location multimapped	0	0	0	0	0	‍0
	Fail, bad location uniquely mapped	0	0	0	0	0	0
	Pass, uniquely mapped Pass, multimapped	0	0	0	0	0	‍0
1500	Pass, uniquely mapped	18,541	18,073	18,163	17,794	72,571	‍90.71
	Pass, multimapped	1459	1927	1837	2206	7429	9.29
	Fail, bad location multimapped	0	0	0	0	0	‍0
	Fail, bad location uniquely mapped	0	0	0	0	0	0
	Pass, uniquely mapped Pass, multimapped	0	0	0	0	0	‍0
2000	Pass, uniquely mapped	18,802	18,448	18,524	18,134	73,908	‍92.38
	Pass, multimapped	1198	1552	1476	1866	6092	7.62
	Fail, bad location multimapped	0	0	0	0	0	‍0
	Fail, bad location uniquely mapped	0	0	0	0	0	0
	Pass, uniquely mapped Pass, multimapped	0	0	0	0	0	‍0
2500	Pass, uniquely mapped	18,993	18,784	18,827	18,507	75,111	‍93.89
	Pass, multimapped	1007	1216	1173	1493	4889	6.11
	Fail, bad location multimapped	0	0	0	0	0	‍0
	Fail, bad location uniquely mapped	0	0	0	0	0	0
	Pass, uniquely mapped Pass, multimapped	0	0	0	0	0	‍0
5000	Pass, uniquely mapped	19,589	19,537	19,580	19,270	77,976	97.47
	Pass, multimapped	411	463	420	730	2024	2.53
	Fail, bad location multimapped	0	0	0	0	0	0
	Fail, bad location uniquely mapped	0	0	0	0	0	0
	Pass, uniquely mapped Pass, multimapped	0	0	0	0	0	0
10,000	Pass, uniquely mapped	19,801	19,843	19,848	19,342	78,834	98.55
	Pass, multimapped	199	157	152	658	1166	1.45
	Fail, bad location multimapped	0	0	0	0	0	0
	Fail, bad location uniquely mapped	0	0	0	0	0	0
	Pass, uniquely mapped	0	0	0	0	0	0
	Pass, multimapped	0	0	0	0	0	0

The length of the flanking sequence on either side of the selected nucleotide position (herein referred to as the ‘marker’) was set to 50, 100, 200, 500, 1,000, 1,500, 2,000, 2,500, 5,000 or 10,000 bp. The aim was to compare the performance of the pipeline across this variable.

A total of 80,000 single nucleotide positions were randomly selected on all seven *Pisum sativum* chromosomes and unassembled scaffolds. The nucleotide positions, herein referred to as markers, were evenly selected in untranslated regions (UTRs), coding sequences (CDSs), introns or repeated elements. This table illustrates the number of markers falling in each alignment category based on their respective genomic positions for different flanking sequence lengths considered, ranging from 50 to 10,000 base pairs (bp) on each side of the selected nucleotide position.

After filtering based on the alignments, each marker was given one of the following statuses:

- Pass, uniquely mapped: A single correct alignment with all filters passed, and the resulting genomic position matches the expected position.
- Pass, multimapped: Multiple valid alignments remain after filtering, mapping to different genomic positions. At least one alignment corresponds to the expected position.
- Fail, bad location, multimapped: Multiple valid alignments remain after filtering, mapping to different genomic positions, but none correspond to the expected position.
- Fail, bad location, uniquely mapped: A single valid alignment is found, but it does not match the expected position.
- Unaligned: No valid alignment is found, most likely due to low complexity of the context sequence.

**Fig. 2:**
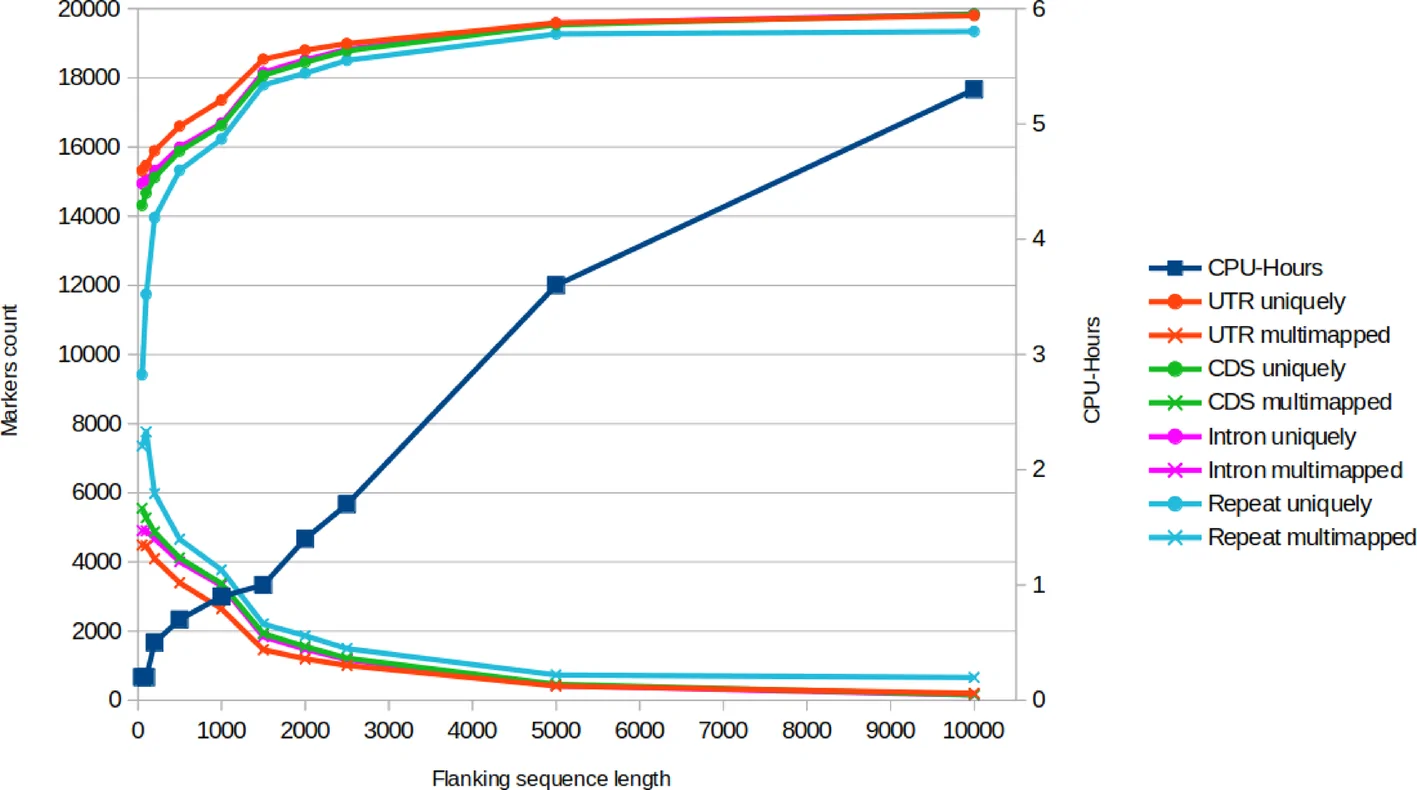
Effect of flanking sequence length on marker alignment performance and computational time

Considering all genomic categories (UTR, CDS, Intron, and Repeat), increasing the length of the context sequence progressively improved marker repositioning, while also increasing computational time (Table 1, Fig. 2). The number of correctly positioned markers (pass) increased from 53,981 uniquely mapped sequences and 22,284 multimapped sequences with 50 bp flanking sequences to, 75,111 uniquely mapped sequences and 4,889multimapped sequences with 2,500 bp flanking sequences, representing a 39.14% increase in uniquely mapped sequences. At the same time, the number of positioning errors (fail, bad location on genome) drops sharply from 2810 to 0 markers. Unaligned markers remain rare and constant across different context sequence lengths, suggesting that the lack of alignment is due to the specific characteristics of the sequence rather than their length. As illustrated in Fig. 2, improvements in mapping specificity are accompanied by a progressive increase in computational time as the length of the flanking sequenceincreases.

Variation in the number of correctly positioned markers and computational time as a function of flanking sequence length (bp). A total of 80,000 randomly selected genomic positions were distributed across untranslated regions (UTRs), coding sequences (CDSs), introns, and repetitive regions. The blue curve shows the CPU-Hours (, right y-axis). Increasing the length of the flanking sequence progressively improves alignment accuracy, resulting in a higher proportion of uniquely mapped markers and fewer in multimapped markers, but also increases computational cost.

Comparisons across genomic categories show that UTRs, CDS, and introns exhibit similar trends with increasing total context sequence length (Table 1). In these three categories, the number of correctly positioned markers (‘Pass, uniquely mapped’) increases progressively, while multimapping decreases comparably. The proportions of correctly aligned markers are similar for a given flanking sequence length. At 50 bp, 76.58% of UTRs, 71.56% of CDS, and 74.7% of introns are correctly positioned when considering uniquely mapped markers, reaching 97,95%, 97.69%, and 97.9%, respectively, at 5,000 bp. When considering both correctly positioned markers (uniquely and multimapped), performance reaches saturation rapidly, with 100% of markers correctly positioned from 500 bp onwards for UTRs and from 1,000 bp onwards for the other categories. However, increasing the context sequence length continues to improve the proportion of uniquely mapped markers, thereby reducing multimapping.

In contrast, repetitive regions display a distinct profile. When considering uniquely mapped markers only, they show an initially lower proportion of pass, uniquely mapped markers (47.1% at 50 bp), with a strong improvement as flanking sequence length increases, reaching 97.5% at 5000 bp. When considering both uniquely and multimapped markers, the proportion of correctly positioned markers is already high but remains lower than in UTRs, CDS, and introns at short flanking lengths, reaching approximately 84% at 50 bp, before increasing to 100% at 1,000 bp,indicating saturation at the same flanking sequence length as other genomic categories. As observed for UTRs, CDS, and introns, increasing the context sequence length primarily improves mapping specificity by increasing the proportion of uniquely mapped markers and reducing multimapping.

This difference can be attributed to the repetitive nature of these regions, which creates greater alignment ambiguities when the context sequence is short. These ambiguities are progressively reduced as longer flanking sequences increase sequence specificity and improve mapping resolution.

Thus, while UTRs, CDS, and introns show comparable and relatively stable behavior, repeat-rich regions are much more sensitive to total context sequence length and benefit more from its increase.

Overall, increasing flanking sequence length does not substantially change the total number of correctly positioned markers, which rapidly reaches saturation (~ 100% from ~ 1,000 bp onward). In contrast, it mainly improves mapping specificity by increasing the proportion of uniquely mapped markers and reducing multimapping. At short flanking lengths (~ 50 bp), performance is already high (> 95% correctly positioned markers), featuring the robustness of AlignMarkers for short sequences.

Although longer flanking sequences increase computational time, they are particularly beneficial in repeat-rich regions. In this example, a flanking sequence length of around 2000 bp provides a practical compromise between computational cost and mapping specificity (Fig. 2).

### Example application 2 comparison of CrossMap, Bcftools/Liftover and AlignMarkers performance using SNPs and indels derived from 100,000 *Solanum lycopersicum* markers

To compare the performance of CrossMap, Bcftools/liftover, and AlignMarkers, we used a randomly selected subset of 100,000 markers extracted from the Varitome whole-genome SNP dataset (*varitome_all.vcf*), available through the Sol Genomics Network (SGN) [39] for *Solanum lycopersicum* (tomato) (~ 850 Mb). This subset comprised 50,000 SNPs and 50,000 indels.

Based on the results obtained in Example application 1, the performance of the AlignMarkers pipeline was assessed using a flanking sequence context of 2000 bp upstream and downstream of each selected nucleotide position, hereafter referred to as a “marker” The genomic distribution of the selected markers across chromosomes is summarized in Supplementary Table 2. Position transfer was performed from the *S_lycopersicum_chromosomes.2.50* reference genome assembly [39] to the *S_lycopersicum_chromosomes.4.00* reference genome assembly [39]. A chain file was produced between those two genomes by aligning them using Minimap2 with the options “ -cx asm5 –cs” and paf2chain script. The chain file produced was then sorted using chainSort.

CrossMap successfully transferred 49,615 SNPs (99.23%) and 49,642 indels (99.28%), with a runtime of approximately ten seconds whereas chain file generation required 40 s to compute the index for Minimap2 and 8 min and 40 s (0.30 CPU-hours) for alignment. Bcftools/Liftover transferred 49,685 SNPs (99.37%) and 49,688 indels (99.37%), also with a runtime of approximately ten seconds. Between these two chain-based tools, 49,615 SNPs and 48,376 indels shared identical genomic positions. Among the variants not mapped by CrossMap, Bcftools/Liftover successfully recovered an additional 59 SNPs and 46 indels.

Using 2,000 bp flanking sequences, the AlignMarkers process took approximately 52 min (1.8 CPU hours) to complete. This time includes the time needed for the indexing phase (35 s), since the current version of AlignMarkers relies on the indexing performed by Minimap2 prior to mapping, and does not involve any pre-computing of the index. Indels were represented by two sequences per marker to capture both allelic states explicitly during alignment, resulting in a longer runtime compared to datasets containing only SNPs.

AlignMarkers, uniquely aligned 48,625 SNPs (97.25%), excluding the 202 SNPs multimapped and 1,173 SNPs were classified as either “bad position” or “bad variation». For indels, AlignMarkers uniquely aligned 46,861 markers (93.72%), while the remaining markers corresponded to multimapping cases.

**Fig. 3:**
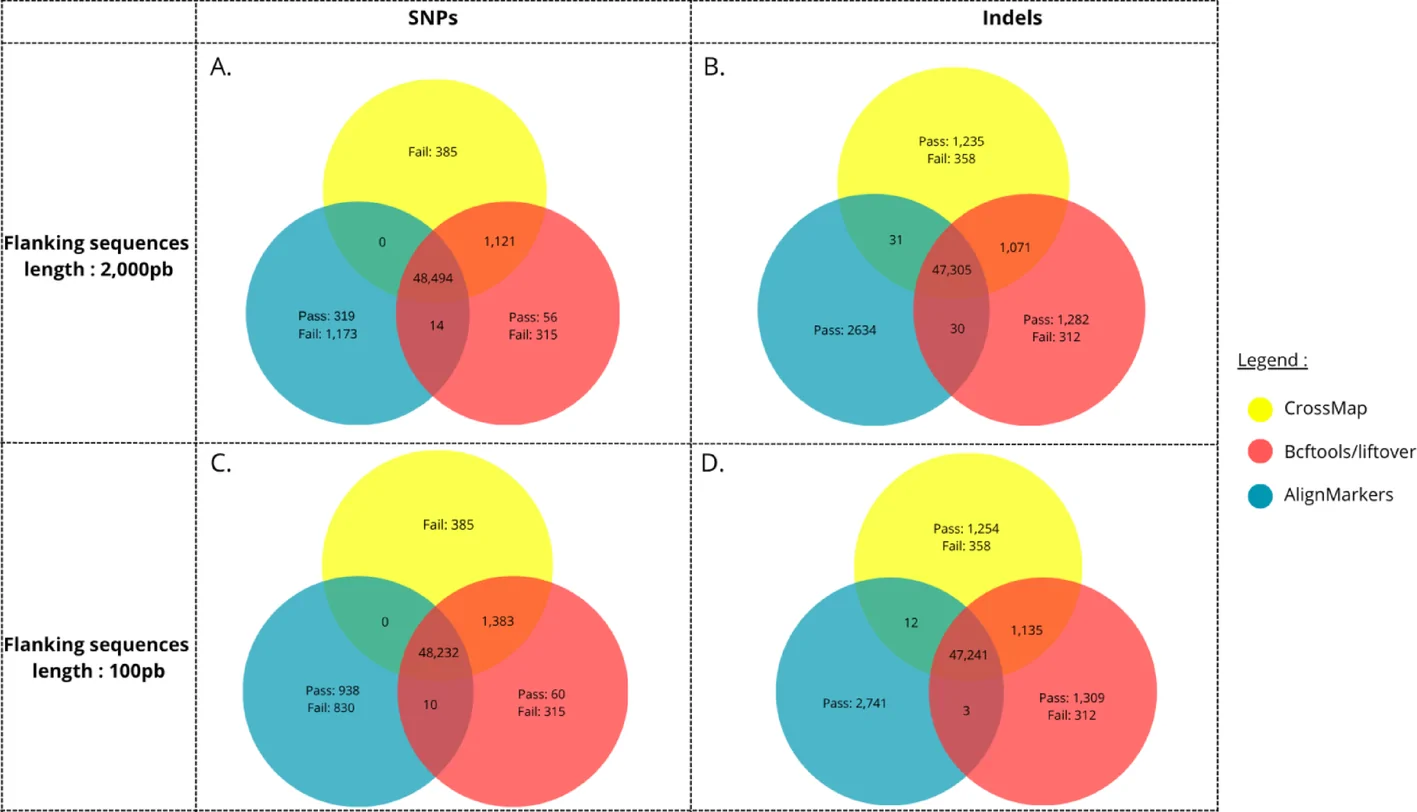
Venn diagrams showing the number of markers whose positions are identical after transfer from *Solanum lycopersicum* 2.50 reference genome assembly to *S. lycopersicum* 4.00 reference genome assembly, using three bioinformatics tools: CrossMap, Bcftools/liftover and AlignMarkers

A total of 48,494 SNPs (96.98%) and 47,305 indels (94.61) were consistently mapped to identical genomic positions across the three tools: CrossMap, Bcftools/Liftover, and AlignMarkers (Fig. 3.A and 3.B).

When comparing AlignMarkers and CrossMap, 48,494 SNPs (96.98%) showed concordant genomic positions between the two tools. This included 48,311 SNPs identified uniquely by AlignMarkers, as well as 180 of the 202 SNPs classified as multimapped that also matched the positions reported by CrossMap. Among the 385 SNPs unmapped by CrossMap, 226 were uniquely and correctly aligned by AlignMarkers. For indels, 47,336 markers (94.67%) showed concordant genomic positions between AlignMarkers and CrossMap. Among these, 45,487 were uniquely mapped and 1,849 were multimapped by AlignMarkers. Overall, AlignMarkers aligned a total of 46,861 indels (93.72%) uniquely. Among the 358 indels unmapped by CrossMap, 70 were successfully and correctly aligned by AlignMarkers.

Comparisons between Bcftools/Liftover and AlignMarkers showed that 48,325 uniquely mapped SNPs shared identical positions, together with 187 multimapped SNPs. For indels, 45,473 uniquely mapped and an additional 1862 multimapped markers were concordant between both tools.

To further evaluate the robustness of AlignMarkers with shorter context sequences, analyses were repeated using 100 bp flanking regions on each side of the markers, corresponding to a total context size of 201 bp. AlignMarkers required approximately 19 min (0.9 CPU-hours) to run. Under these conditions, 48,232 SNPs were concordantly positioned across the three tools, including 45,016 uniquely mapped and 3216 multimapped markers. For indels, 47,241 markers were shared across all tools, including 42,107 uniquely mapped and 5134 multimapped markers (Fig. 3.C and 3.D).

In the event of multimapping, AlignMarkers should indicate the true position as its first hit. To evaluate this, the first alignment proposed by the pipeline for the multimapped markers was compared with the positions identified by CrossMap and BCFtools/Liftover.

For indels, CrossMap recovered the same first alignment as AlignMarkers in 99.42% of multimapped cases when using 100 bp flanking regions, and in 98.67% of cases with 2000 bp flanking regions. BCFtools/Liftover showed slightly lower concordance rates, with 98.96% and 98.15% agreement for 100 bp and 2000 bp flanking regions, respectively.

For SNPs, CrossMap matched the first AlignMarkers alignment in 99.25% of multimapped cases with 100 bp flanking regions and 97.9% with 2000 bp flanking regions. Corresponding concordance rates for BCFtools/Liftover were 98.8% and 97.3%, respectively.

The analyses are presented according to variant type (SNPs or indels) and the length of the flanking sequences used.

A. SNPs with 2000 bp flanking sequences on each side
B. Indels with 2000 bp flanking sequences on each side
C. SNPs with 100 bp flanking sequences on each side
D. Indels with 100 bp flanking sequences on each side

The intersections indicate the number of markers shared by the three methods, while the non-overlapping regions represent markers with positions specific to each tool.

The results obtained with 100 bp flanking sequences were highly similar to those obtained with 2000 bp flanking sequences, demonstrating that AlignMarkers is capable of accurately repositioning markers even when using relatively short sequence contexts. The main difference was an increase in the number of markers classified as multimapped by the pipeline when shorter sequences were used. Overall, these results highlight the strong concordance between AlignMarkers and established lift-over tools while also showing that AlignMarkers could successfully recover additional variants missed by chain-based approaches, and provide useful information on non-unique context sequences, which is useful for specific hybridization-based applications. Although longer flanking sequences improve mapping specificity, short sequence contexts already provide reliable marker repositioning results.

### Example application 3 considering 1920 expressed sequence tag-derived SNPs selected for *Pisum sativum* genetic diversity assessment

The results obtained in Example applications 1 and 2 demonstrated that AlignMarkers can accurately transfer molecular marker positions even when only short flanking sequences are used. To further assess this capability on transcriptome-derived markers associated with very short sequence contexts, AlignMarkers was applied on a set of 1,920 SNPs identified from the transcriptomes of eight pea genotypes representative of modern European breeding material, including winter and spring types [40]. These markers, along with their 201 bp context sequences, were aligned to the pea Cameor V2 reference genome [38].

Using default Minimap2 parameters, 1433 markers (74.6%) successfully passed all filtering criteria and were considered properly placed.

Because these SNPs were derived from transcript sequences and provided context sequences may span introns, the -x splice option of Minimap2 was applied. This option enables splice-aware alignment by treating long deletions as introns. When applied to the entire dataset, 1677 markers (87.3%) passed the filters. However, in this global splice run, 24 markers that had passed under default settings failed the filters, with 6 now considered multimapped, 2 showing insufficient identity percentage, and 16 classified as ‘bad position’. This observation suggests that a conditional strategy is more appropriate, applying the -x splice option only to markers that failed to align properly in the initial run with default parameters.

We therefore restricted the -x splice option to the 487 markers that failed placement with default parameters which allowed for the recovery of 268 additional markers.

Among the remaining 219 markers, 83 were multimapped and 136 were exhibited bad positioning, which means that polymorphism is not in the expected position within the context sequence. Within this last group, 30 markers could pass the filters if truncated alignments were considered, with alignments starting between positions 1 and 98 bp of the query sequence (qstart). And, 18 markers were extremely truncated, covering only positions 102–201 and missing the expected polymorphism at position 101.

Overall, combining the default and conditional splice strategies, 1701 of the 1920 SNP markers (89.1%) were successfully placed with high confidence. This example demonstrates that EST-derived transcript sequence markers can be effectively aligned using short context sequences, provided that alignment parameters are appropriately adapted to their structural characteristics, in particular through splice-aware mapping. In this context, a conditional strategy further improves marker recovery while limiting the risk of misalignment or excessive filtering.

## Conclusion

AlignMarkers was developed to enable reliable transfer of molecular markers (SNPs and indels) to a target genome by sequence alignment, in the context of multi-species genotyping arrays design. The tool supports both coordinate-based and sequence-based inputs, with or without prior access to a reference genome, and is compatible with whole-genome and transcriptomic datasets.

A comparison with CrossMap and Bcftools/Liftover showed that these chain file–based methods mapped a higher proportion of markers and achieved faster runtimes. However, these approaches rely solely on precomputed chain file alignment and do not explicitly assess the quality of alignment in the vicinity of the marker. In contrast, AlignMarkers uses Minimap2 CIGAR to run the alignment, which permits the application of strict filters around the marker for multimapping, poor positioning, unexpected variations, and low sequence identity. Although these filters reduce the total number of successfully mapped markers and increase computational time, they guarantee high-quality output, suitable to build robust amplicons for hybridization-based approaches.

Overall, CrossMap and Bcftools/liftover are well suited for rapid, large-scale coordinate conversion when reliable chain files are available. Conversely, AlignMarkers provides a stringent alternative when alignment accuracy, variant integrity or the absence of suitable chain files are critical constraints. In this case, context sequences with 1000 to 2500 bp on each side of the marker currently provide a practical compromise between computational efficiency and mapping specificity. AlignMarkers can be applied as a coordinate transfer tool between genome assemblies in a manner similar to CrossMap, particularly in cases involving complex or large reference genomes where chain file generation is computationally expensive or infeasible due to the absence of the original reference. While the current implementation is not optimized for long context sequences, future versions will address this limitation through performance improvements and better strategies to limit false-positives for multimapped regions.

Finally, by combining automated retrieval of context sequences, when required, with stringent alignment filtering, AlignMarkers provides accurate marker positioning even in large and heterogeneous datasets. Its modular implementation in Nextflow and Python, integrating widely used bioinformatics tools, ensures both accessibility and robustness. The pipeline accommodates diverse input formats, facilitating marker transfer across genome assemblies and making it suitable for applications such as genotyping tool development, comparative genomics, marker-assisted selection and genomic selection.

## Supplementary Information

Below is the link to the electronic supplementary material.

Supplementary Material 1

## Data Availability

The source code and a user’s manual are freely available at https://forge.inrae.fr/geapsi/pipeline/specifics_alignmarkers. Demo datasets associated with the three example applications described in this study, including the 80,000-marker *Pisum sativum* dataset, the 100,000-marker Solanum lycopersicum SNP/indel dataset, the 1920 EST-derived P. sativum SNP dataset, as well as the corresponding chain files and example outputs, are available at https://entrepot.recherche.data.gouv.fr/privateurl.xhtml?token=54c74594-cfde-4baf-ae0b-2cd0523095fc. The repository also provides the results of the comparative analyses performed with CrossMap, BCFtools/liftover and AlignMarkers.

## Abbreviations

CIGAR: Compact ideosyncratic gapped alignment report
EST: Expressed sequence tag
Indel: Insertion-deletion
PAF: Pairwise mapping format
SNP: Single nucleotide polymorphism

## References

[1] Schwacke R, Bolger ME, Usadel B. PubPlant—a continuously updated online resource for sequenced and published plant genomes. Front Plant Sci. 2025. 10.3389/fpls.2025.1603547. PMC12235464 40630734

[2] Akintunde O, Tucker T, Carabetta VJ. The evolution of next-generation sequencing technologies. ArXiv. 15 May 2023; arXiv:2305.08724v1. PubMed PMID: 37292469; PubMed Central PMCID: PMC10246072. 10.1007/978-1-0716-4192-7_1 39546194

[3] NCBI, Genome. Available in: https://www.ncbi.nlm.nih.gov/datasets/genome/.

[4] Vaseeharan B, Rajakamaran P, Jayaseelan D, Vincent AY. Molecular markers and their application in genetic diversity of penaeid shrimp. Aquac Int. 2013;21(2):219–41. 10.1007/s10499-012-9582-9.

[5] Zhao H, Sun Z, Wang J, Huang H, Kocher JP, Wang L. CrossMap: a versatile tool for coordinate conversion between genome assemblies. Bioinformatics. 2014;30(7):1006–7. 10.1093/bioinformatics/btt730. PMC3967108 24351709

[6] Genovese G, Rockweiler NB, Gorman BR, Bigdeli TB, Pato MT, Pato CN, et al. BCFtools/liftover: an accurate and comprehensive tool to convert genetic variants across genome assemblies. Bioinformatics. 2024;40(2):btae038. 10.1093/bioinformatics/btae038. PMC10832354 38261650

[7] Fujita PA, Rhead B, Zweig AS, Hinrichs AS, Karolchik D, Cline MS, et al. The UCSC Genome Browser database: update 2011. Nucleic Acids Res. 2011;39(suppl_1):D876–82. 10.1093/nar/gkq963. PMC3242726 20959295

[8] Genovese G, Rockweiler NB, Gorman BR, Bigdeli TB, Pato MT, Pato CN, et al. BCFtools/liftover: an accurate and comprehensive tool to convert genetic variants across genome assemblies. Bioinformatics. 2024;40(2):btae038. 10.1093/bioinformatics/btae038. PMC10832354 38261650

[9] Kent WJ, Baertsch R, Hinrichs A, Miller W, Haussler D. Evolution’s cauldron: duplication, deletion, and rearrangement in the mouse and human genomes. Proc Natl Acad Sci U S A. 2003;100(20):11484–9. 10.1073/pnas.1932072100. PMC208784 14500911

[10] Li H, Handsaker B, Wysoker A, Fennell T, Ruan J, Homer N, et al. The sequence Alignment/Map format and SAMtools. Bioinformatics. 2009;25(16):2078–9. 10.1093/bioinformatics/btp352. PMC2723002 19505943

[11] Cochrane G, Alako B, Amid C, Bower L, Cerdeño-Tárraga A, Cleland I, et al. Facing growth in the European nucleotide archive. Nucleic Acids Res. 2013;41(D1):D30–5. 10.1093/nar/gks1175. PMC3531187 23203883

[12] Kent WJ, Sugnet CW, Furey TS, Roskin KM, Pringle TH, Zahler AM, et al. The human genome browser at UCSC. Genome Res. 2002;12(6):996–1006. 10.1101/gr.229102. PMC186604 12045153

[13] Reese MG, Moore B, Batchelor C, Salas F, Cunningham F, Marth GT, et al. A standard variation file format for human genome sequences. Genome Biol. 2010;11(8):R88. 10.1186/gb-2010-11-8-r88. PMC2945790 20796305

[14] GTF2: Mouse/Human Annotation Collaboration: http://mblab.wustl.edu/GTF2.html.

[15] Danecek P, Auton A, Abecasis G, Albers CA, Banks E, DePristo MA, et al. The variant call format and VCFtools. Bioinformatics. 2011;27(15):2156–8. 10.1093/bioinformatics/btr330. PMC3137218 21653522

[16] Talenti A, Prendergast J. Nf-LO: a scalable, containerized workflow for genome-to-genome lift over. Genome Biol Evol. 2021;13(9):evab183. 10.1093/gbe/evab183. PMC8412297 34383887

[17] Guarracino A. AndreaGuarracino/paf2chain: https://github.com/AndreaGuarracino/paf2chain.

[18] Wei W, Gui S, Yang J, Garrison E, Yan J, Liu HJ. Wgatools: an ultrafast toolkit for manipulating whole-genome alignments. Bioinformatics. 2025;41(4):btaf132. 10.1093/bioinformatics/btaf132. PMC11978383 40152239

[19] DoBlastzChainNet.pl—genomewiki: https://genomewiki.ucsc.edu/index.php?title=DoBlastzChainNet.pl.

[20] Luu PL, Ong PT, Dinh TP, Clark SJ. Benchmark study comparing liftover tools for genome conversion of epigenome sequencing data. NAR Genom Bioinform. 2020;2(3):lqaa054. 10.1093/nargab/lqaa054. PMC7671393 33575605

[21] Park KJ, Yoon YA, Park JH. Evaluation of liftover tools for the conversion of genome reference consortium human build 37 to build 38 using ClinVar variants. Genes. 2023;14(10):1875. 10.3390/genes14101875. PMC10606611 37895222

[22] Lan D, Purnomo G, Tobler R, Souilmi Y, Llamas B. Genozip Dual-Coordinate VCF format enables efficient genomic analyses and alleviates liftover limitations: https://www.biorxiv.org/content/10.1101/2022.07.17.500374v2 10.1101/2022.07.17.500374

[23] Genome Browser User’s Guide: https://genome.ucsc.edu/goldenPath/help/hgTracksHelp.html.

[24] Genome Browser FAQ: https://genome.ucsc.edu/FAQ/FAQreleases.html#snpConversion.

[25] Lipman DJ, Pearson WR. Rapid and sensitive protein similarity searches. Science. 1985;227(4693):1435–41. 10.1126/science.2983426. 2983426

[26] Li H. Minimap2: pairwise alignment for nucleotide sequences. Bioinformatics. 2018;34(18):3094–100. 10.1093/bioinformatics/bty191. PMC6137996 29750242

[27] Di Tommaso P, Chatzou M, Floden EW, Barja PP, Palumbo E, Notredame C. Nextflow enables reproducible computational workflows. Nat Biotechnol. 2017;35(4):316–9. 10.1038/nbt.3820. 28398311

[28] Quinlan AR, Hall IM. BEDTools: a flexible suite of utilities for comparing genomic features. Bioinformatics. 2010;26(6):841–2. 10.1093/bioinformatics/btq033. PMC2832824 20110278

[29] Python.org: https://www.python.org/.

[30] Kurtzer GM, Sochat V, Bauer MW. Singularity: scientific containers for mobility of compute. PLoS ONE. 2017;12(5):e0177459. 10.1371/journal.pone.0177459. PMC5426675 28494014

[31] Biopython · Biopython: https://biopython.org/.

[32] Polars: https://www.pola.rs/.

[33] Matplotlib—visualization with Python: https://matplotlib.org/.

[34] R: The R Project for Statistical Computing: https://www.r-project.org/.

[35] UpSet (Venn diagrams alternative) plots extensible with ggplot2 geoms: https://krassowski.github.io/complex-upset/.

[36] OpenAI. Version GPT-5.1. San Francisco (CA): OpenAI; 2025: https://openai.com/fr-FR/.

[37] IFB Core Cluster Documentation: https://doc.cluster.france-bioinformatique.fr/.

[38] Kreplak J, Novak P, Robledillo LÁ, Aubert G, Imbert B, Kaur P, et al. A new genome assembly of the pea cultivar ‘Caméor’ provides resources for functional genomics and genetics: https://www.biorxiv.org/content/10.1101/2025.04.01.645976v1 10.1101/2025.04.01.645976 PMC13389026 42120873

[39] Sol Genomics Network: https://solgenomics.net/projects/varitome.

[40] Duarte J, Rivière N, Baranger A, Aubert G, Burstin J, Cornet L, et al. Transcriptome sequencing for high throughput SNP development and genetic mapping in pea. BMC Genomics. 2014;15(1):126. 10.1186/1471-2164-15-126. PMC3925251 24521263

