# Supplementary material for: AlignMarkers: an original pipeline for placing molecular markers on physical sequences
Source: BMC Bioinformatics. 2026 Aug 6;27:266. doi: 10.1186/s12859-026-06582-1 (PMC13746528; doi:10.1186/s12859-026-06582-1)
Supplement: Supplementary file 1 [file 12859_2026_6582_MOESM1_ESM.docx]

**SUPPLEMENTARY**

**Supplementary table 1.** Distribution of the 80,000 randomly selected markers in the *Pisum sativum* Cameor V2 genome assembly by chromosome and genomic category

Each genomic category (UTR, CDS, Intron, and Repeat) is represented by 20,000 randomly selected markers. The table shows the number of markers per chromosome and per category. All markers located on scaffolds were counted together.

| **Chromosome/Scaffold** | **UTR** | **CDS** | **Intron** | **Repeat** | **TOTAL** |
| --- | --- | --- | --- | --- | --- |
| ‍Chr1 | 2748 | 2530 | 2553 | 2467 | 10298 |
| ‍Chr2 | 2399 | 2206 | 2297 | 2551 | 9453 |
| ‍Chr3 | 2539 | 2540 | 2536 | 2714 | 10329 |
| ‍Chr4 | 2692 | 2786 | 2853 | 2735 | 11066‍ |
| Chr5 | 3571 | 3683 | 3720 | 3377 | 14351 |
| ‍Chr6 | 2804 | 2886 | 2851 | 2777 | 11318 |
| ‍Chr7 | 2957 | 3079 | 2904 | 2856 | 11796 |
| ‍Scaffold | 290 | 290 | 286 | 523 | 1389 |
| ‍TOTAL | 20000 | 20000 | 20000 | 20000 | 80000 |

**Supplementary table 2.** Distribution of the 100,000 randomly selected markers from the *Solanum lycopersicum* VCF file by chromosome and markers type.

| Chromosome | Number of SNPs | Number of Indels |
| --- | --- | --- |
| Chr00 | 895 | 735 |
| Chr01 | 4720 | 5206 |
| Chr02 | 2805 | 3371 |
| Chr03 | 3792 | 4412 |
| Chr04 | 4768 | 4685 |
| Chr05 | 3689 | 3122 |
| Chr06 | 3265 | 2966 |
| Chr07 | 5285 | 6124 |
| Chr08 | 5264 | 5140 |
| ‍Chr09 | 3262 | 3150 |
| ‍Chr10 | 3652 | 3504 |
| ‍Chr11 | 3794 | 4237 |
| ‍Chr12 | 4269 | 3888 |
